# IL-6 Signaling in Myelomonocytic Cells Is Not Crucial for the Development of IMQ-Induced Psoriasis

**DOI:** 10.1371/journal.pone.0151913

**Published:** 2016-03-21

**Authors:** Sabrina Klebow, Matthias Hahn, Alexei Nikoalev, F. Thomas Wunderlich, Nadine Hövelmeyer, Susanne H. Karbach, Ari Waisman

**Affiliations:** 1 Institute for Molecular Medicine, University Medical Center of the Johannes-Gutenberg University of Mainz, Mainz, Germany; 2 Max Planck Institute for Metabolism Research, Cologne, Germany; 3 Department of Medicine 2, University Medical Center of the Johannes-Gutenberg University of Mainz, Mainz, Germany; French National Centre for Scientific Research, FRANCE

## Abstract

Psoriasis is an autoimmune skin disease that is associated with aberrant activity of immune cells and keratinocytes. In mice, topical application of TLR7/8 agonist IMQ leads to a skin disorder resembling human psoriasis. Recently, it was shown that the IL-23/ IL-17 axis plays a deciding role in the pathogenesis of human psoriasis, as well as in the mouse model of IMQ-induced psoriasis-like skin disease. A consequence of IL-17A production in the skin includes increased expression and production of IL-6, resulting in the recruitment of neutrophils and other myelomonocytic cells to the site of inflammation. To further investigate and characterize the exact role of IL-6 signaling in myelomonocytic cells during experimental psoriasis, we generated mice lacking the IL-6 receptor alpha specifically in myelomonocytic cells (*IL-6Rα*^*Δmyel*^). Surprisingly, disease susceptibility of these mice was not affected in this model. Our study shows that classical IL-6 signaling in myelomonocytic cells does not play an essential role for disease development of IMQ-induced psoriasis-like skin disease.

## Introduction

Psoriasis is a chronic autoimmune skin disease affecting up to 2–4% of the worldwide population [1]. It develops over time, mostly in late adolescence or early adulthood [2] and is dependent on a complex interaction of different genetic and environmental factors [3]. The common form is psoriasis vulgaris and comes along with red, scaly plaques in certain areas (knees, elbows and the scalp) [2]. Since psoriasis can be treated successfully with immunosuppressive drugs like cyclosporine, it is clear that the immune system has a pathogenic role in the development of this disease [3]. Newer, more specific treatment options consist in monoclonal antibodies against TNF-α [4] or Interleukin (IL)-17 [5]. The immunopathogenesis of psoriasis is based on a strong interplay between different immune cells and keratinocytes [1]. In the epidermis of psoriatic lesions keratinocytes proliferate abnormally which leads to the formation of scaly plaques [2]. Furthermore, neutrophils build small foci in the stratum corneum and mononuclear leukocytes, like T cells and dendritic cells, infiltrate into the dermis [2].

A common mouse model of psoriasis like disease is induced by application of 5% imiquimod (IMQ)—containing *Aldara* cream to the mouse skin [6]. IMQ is a toll—like receptor (TLR) 7/8 agonist and an immune cell activator [7]. The application of IMQ to mouse skin leads to dermal damage comparable to that seen in human psoriasis [6].

The IL-23/ IL-17 axis is important in the development of human psoriasis and in IMQ-induced psoriasis-like skin disease [3,6]. The number of IL-17 producing cells is increased in the skin of mice treated with IMQ [6,8,9]. There is clear evidence, that the main producers of IL-17 in IMQ-induced psoriasis-like skin disease are the γδ T cells [6,8,10]. Within these, dermal and epidermal γδ T cells can be distinguished whereas the main producers of IL-17A in IMQ-induced psoriasis are the dermal γδ T cells [8,10]. In line with these data, we could already show that mice with a deletion of IL-17RA still develop psoriasis—like disease under IMQ treatment, but the disease was milder compared to wild type mice [9].

Injection of IL-23, which is up-stream of IL-17 signaling [11], leads to erythema and inflammatory infiltrates [12,13]. Interestingly, effects of IL-23 are milder in *IL6*^*-/-*^ mice. Here, the injection of IL-23 induces an attenuated skin disease [14], suggesting a strong involvement of IL-6.

As IL-6 is important in the pathogenesis of psoriasis, anti-IL-6 has been discussed as a new treatment option of psoriasis in humans [15], but stays disputed. Treatment with tocilizumab, an anti-IL-6R antibody, has been described to be helpful in other autoimmune diseases, like rheumatoid arthritis [16,17] or even psoriatic arthritis [18]. It remains controversial that tocilizumab treated rheumatoid arthritis patients can also develop psoriasis during the treatment [19,20].

IL-6 is a pleiotropic cytokine that occurs in two different forms: classical signaling and trans-signaling with the soluble form of the IL-6Rα (sIL-6Rα). Only cells that express the membrane bound IL-6Rα (mIL-6Rα) can respond to IL-6 in the classical way, whereas trans-signaling affects nearly every cell type when expressing the co-receptor molecule gp130 [21]. In humans, the sIL-6Rα is generated by proteolysis of the metalloproteases ADAM 10 and 17 (90%) or alternative splicing (10%) [22,23].

Recent data suggests that IL-6 plays an important role in the pathogenesis of psoriasis. In the model of IMQ-induced psoriasis-like skin disease, IL-6 is important for recruitment of neutrophils [24]. Furthermore, serum levels of IL-6 are elevated in patients with psoriasis [25] and IL-6 leads to a stronger proliferation of human keratinocytes [26]. During psoriasis, IL-6 is primarily released by keratinocytes. They regulate epidermal hyperplasia and fibroplasia in psoriatic lesions [2]. Our group generated a mouse strain, which develops a skin phenotype with many hallmarks of human psoriasis (*K14-IL-17A*^*ind/+*^) [27]. In this mouse model the cytokine IL-17A is specifically overexpressed in keratinocytes, resulting in a strong psoriasis-like phenotype. The treatment of these mice with an anti-IL-6 antibody led to a reduction of neutrophil micro-abscesses in the skin [27], showing the importance of IL-6 in the context of myelomonocytic cells. Myelomonocytic cells are lysozyme M (LysM) positive cells, such as monocytes, macrophages and neutrophils, expressing the major isoform of lysozyme [28].

To investigate the role of IL-6 signaling in the myelomonocytic cell compartment, we decided to specifically delete the IL-6 receptor alpha (IL-6Rα) in myelomonocytic cells using the LysMCre mouse line (*IL-6Rα*^*Δmyel*^). Surprisingly, the application of IMQ to the mouse skin led to a similar clinical phenotype in *IL-6Rα*^*Δmyel*^ mice and control animals. We failed to detect any differences in the extent of myelomonocytic cell or T cell infiltration in the skin and secondary lymphoid organs, which suggests that sIL-6Rα derived from non-myelomonocytic cells might compensate the loss of classical IL-6R signaling in the myelomonocytic compartment.

## Material and Methods

### Mice

*IL6Rα*^*Δmyel*^ mice were generated by crossing the IL-6Rα allele [29] to the LysM-Cre allele [30]. The mice were bred in the animal facility at the University Medical Center of Mainz. All animal experiments were in accordance with the guidelines of the central animal facility institution (ZVTE, University of Mainz). All mice were on C57BL/6 background and housed in specific-pathogen-free conditions in the animal facility at the University of Mainz. All animal experiments were carried out in accordance with the guidelines of the Central Animal Facility Institution (CLAF, University of Mainz). Mice were euthanized with an overdose of isoflurane. Animal Care and Use Committee (IACUC) from the Land of Rhineland Palatine (RLP) approved all experiments with Permit Number 23 177-07/G13-1-099. All surgery was performed under anesthesia, and all efforts were made to minimize suffering.

### IMQ-induced psoriasis-like skin disease

Female mice at the age of 7–8 weeks were either treated with *Aldara* (5% IMQ; Meda AB, Solna, Sweden) or sham cream [6] on ears (each with 5mg) as well as the back skin (50mg) for 5 consecutive days. 48h before the first treatment with either IMQ or sham cream, back skin of the mice was shaved with an electric shaver and the remaining hair was removed with *Veet* hair removal cream. Mice were treated daily in the late morning and sacrificed 20h after the last treatment. Anesthesia was performed with a mixture of ketamine/xylazine. To prevent a high weight loss due to the daily treatment and anesthesia, mice were injected with 600μl of sodium chloride daily.

### PASI score

To measure the severity of inflammation on the back, a scoring system similar to the human PASI (Psoriasis Area and Severity Index) score was used. In mice, this score considers the parameters of skin thickness, scaling and erythema. A cumulative PASI score or the individual score concerning one parameter (skin thickness, scaling, erythema) is shown. The measurement of the skin thickness was performed in triplicates with a dial thickness gage (Mitutoyo, Kawasaki, Japan).

### Flow Cytometry

Single cell suspensions were prepared from different organs. Prior to the preparation of single cell suspension organs were kept in 1x phosphate buffered saline (PBS) plus 2% (v/v) fetal calf serum (FCS). Ears were kept in only PBS. The spleens were isolated and filtered through a sterile sieve (40μm) to obtain single cell suspensions. Bones were flushed wit a syringe containing PBS plus 2% (v/v) FCS to extract cells from the bone marrow (BM). Red blood cells isolated from spleen and bone marrow, were lysed with tris-ammonium chloride, pH 7.2. Blood was lysed with BD FACS lysing solution (BD Pharmingen). Cells of the skin were digested with 0.25mg/ml liberase TM (Roche) and subsequently homogenized with 0.6mmx30mm needles. Single-cell suspensions were treated with Fc-Block (BioXCell) and surface stained with monoclonal antibodies: Ly6G (BL 1A8), CD90.2 (eBio 53–2.1), B220 (BL RA3-6B2), CD11b (eBio M1/70), F4/80 (eBio BM8), Ly6C (BD AL-21), Gr-1 (BD RB6-8C5), γδTCR (eBio eBioGL3), CD3e (BD 145-2C11), CD45.2 (BD 104), CD19 (BL 6D5, BD 1D3), CD126 (IL-6Rα) (D7715A7). For biotinylated antibodies streptavidin in V500 (BD Pharmingen) or Pe-Cy7 (eBio) was used. Dead cells were excluded with fixable viability dye ef780 (eBio). All samples were acquired with FACS Canto II (BD Pharmingen) and analyzed with FlowJo Version 8.87.

### Intracellular staining

Cells were stimulated in culture medium containing phorbol 12-myristate 13-acetate (50 ng/ml) and ionomycin (500 ng/ml), in the presence of Brefeldin A (1 μg/ml) at 37°C and 5% CO_2_ for 4hrs. After staining the surface markers, cells were fixed and permeabilized (0.1% Saponin and 2% PFA) and followed by staining with an antibody of mouse IL-17A (eBio 17B7).

### Histology

Immunohistochemistry of 8μm skin cryosections was performed using the fluorescence microscope Olympus IX81 (Olympus, Tokyo, Japan) and the TSA Cy3 (PerkinElmer, Waltham, MA) as recommended by the company. The following primary antibodies were used: F4/80 (BD Pharmingen), myeloperoxidase (Abcam, Cambridge, MA). The slides were incubated for 30 minutes at room temperature with the biotinylated secondary antibody (Dianova, Hamburg, Germany; BD). Nuclei were counterstained with Hoechst 33342 (Invitrogen). Tissues were mounted in Vectashield H-1000.

### Cell purification

Cells from bone marrow were purified using anti-CD11b magnetic beads (Milteny Biotech) according to manufacture’s instruction. Purity was determined by flow cytometry and was above 94%.

### RNA isolation and Quantitative Real Time PCR

MACS purified CD11b^+^ bone marrow cells from *IL6ra*^*Δmyel*^ and wildtype (*wt*) mice were used for total RNA isolation using the peqGOLD Total RNA Kit (Peqlab). Quantitative real-time PCR (RT-PCR) was performed with the QuantiTect SYBR Green RT-PCR Kit using primers from Qiagen as described on their homepage: http://www.qiagen.com/products/pcr/quantitect/primerassays.aspx.

All changes in gene expression were calculated relative to that of the house keeping gene hypoxanthine-guanine phosphoribosyltransferase (HPRT).

### ELISA

To detect the cytokine levels of sIL-6Rα, a DuoSet® ELISA from R&D systems was performed. All corresponding antibodies were used from R&D systems. The standard curve demonstrated direct relationship between OD and secreted cytokine levels.

### Statistical analysis

For statistics, Prism® (GraphPad 5 Software Inc.) was used. Values are typically as mean ± SEM (standard error of the mean). For statistical analysis first the Kologorow-Smirnow test was performed to test the normal distribution. If this was given, 2-tailed unpaired student’s *t*-test or 1-way ANOVA were applied. If there was no normal distribution given, Mann-Whitney or Kruskal-Wallis were used as appropriate. P-values < 0.05 were regarded significant, displayed by “*” in the figures (** = p-values < 0.005; *** = p-values < 0.001).

## Results

### Myelomonocytic cell specific deletion of the IL-6Rα

The murine model of psoriasis-like skin disease, induced by application of IMQ to the skin, involves a strong recruitment of neutrophils and other myelomonocytic cells to the site of inflammation [31], similarly to what is observed in human psoriasis. We, and others have shown that IL-6 and its downstream targets (e.g. Stat3) are involved in the pathogenicity of IMQ-induced psoriasis-like skin disease [9] and development of psoriatic lesions [27,32]. IL-6 is of great importance for general neutrophil recruitment [24].

**Fig 1:**
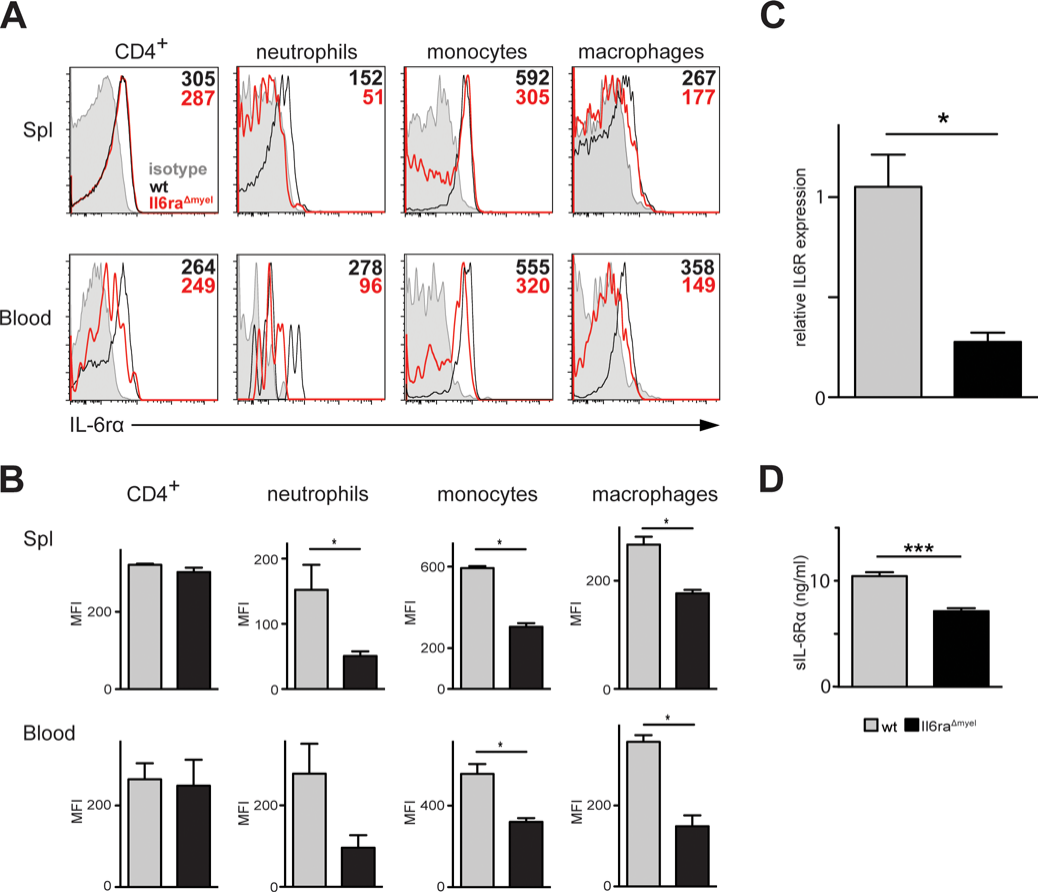
Functionality of *Il6ra*^*Δmyel*^ mice. (A) Flow cytometric analysis of IL-6Rα expression of different organs. CD4^+^ cells are pre-gated on living CD90.2^+^ cells. Neutrophils (Gr-1^hi^ F4/80^-^), monocytes (Gr-1^int^ F4/80^+^) and macrophages (Gr-1^-^ F4/80^+^) are pre-gated on living CD90.2^-^/B220^-^ and CD11b^+^ cells. Gray histograms represent IgG2b isotype control for the IL-6R staining. Numbers in upper right corner represent the mean Mean Fluorescent Intensity (MFI) values of Il6ra^Δmyel^ or *wt* cells. Shown are representative histograms (n = 7 or 8 (3 independent experiments). (B) MFI of IL-6Rα expression pre-gated on CD4^+^ cells, neutrophils (Gr-1^hi^ F4/80^-^), monocytes (Gr-1^int^ F4/80^+^) or macrophages (Gr-1^-^ F4/80^+^). Data are shown as bar graphs with mean and SEM. *p ≤ 0,05 Significance was calculated using Mann Whitney test. (C) Quantitative RT-PCR from CD11b^+^ MACS purified bone marrow cells for the IL-6Rα gene in *wt* and *Il6ra*^*Δmyel*^ mice. Expression levels are shown relative to the housekeeping gene HPRT (n = 5). Data are shown as bar graphs with mean and SEM. * p ≤ 0,05 Significance was calculated using Mann Whitney test. (D) Serum concentrations of sIL-6Rα examined by ELISA in *wt* (n = 6) and *Il6ra*^*Δmyel*^ (n = 10) mice at the age of 5 weeks to 5 months. Data are shown as bar graphs with mean and SEM. *** p ≤ 0,001 Significance was calculated using Mann Whitney test.

To further investigate the role of IL-6 signaling in myelomonocytic cells in the context of IMQ-induced psoriasis-like skin disease, we crossed LysM-Cre mice [30] with the IL-6Rα^FL/FL^ mouse line [29]. This resulted in a mouse line lacking the membrane-bound IL-6Rα (mIL-6Rα) exclusively in myelomonocytic cells (*IL-6Rα*^*Δmyel*^ mice). These mice have a reduced expression of the receptor in the myelomonocytic compartment (neutrophils, monocytes and macrophages) in spleen and blood compared to control mice, shown by flow cytometric analysis, but no difference in IL-6Rα MFI was observed in CD4^+^ cells (Fig 1A and 1B). Moreover, in these mice there is a significant reduction in mIL-6Rα expression in myelomonocytic cells shown by RT-PCR of CD11b^+^ isolated bone marrow cells (Fig 1C). We also found a significant reduction in the systemic levels of sIL-6Rα measured in the serum (Fig 1D) indicating the importance of myelomonocytic cells as source of this molecule. It is a clear hint that IL-6 signaling of the myelomonocytic compartment is necessary for the secretion of sIL-6Rα. In contrast to that, the liver-specific deletion of the IL-6Rα gene had not resulted in decreased levels of the soluble IL-6Rα in the blood as described previously [29].

### Deletion of IL-6Rα in myelomonocytic cells does not result in major differences in immune cell populations in spleen, blood and bone marrow at baseline

**Fig 2:**
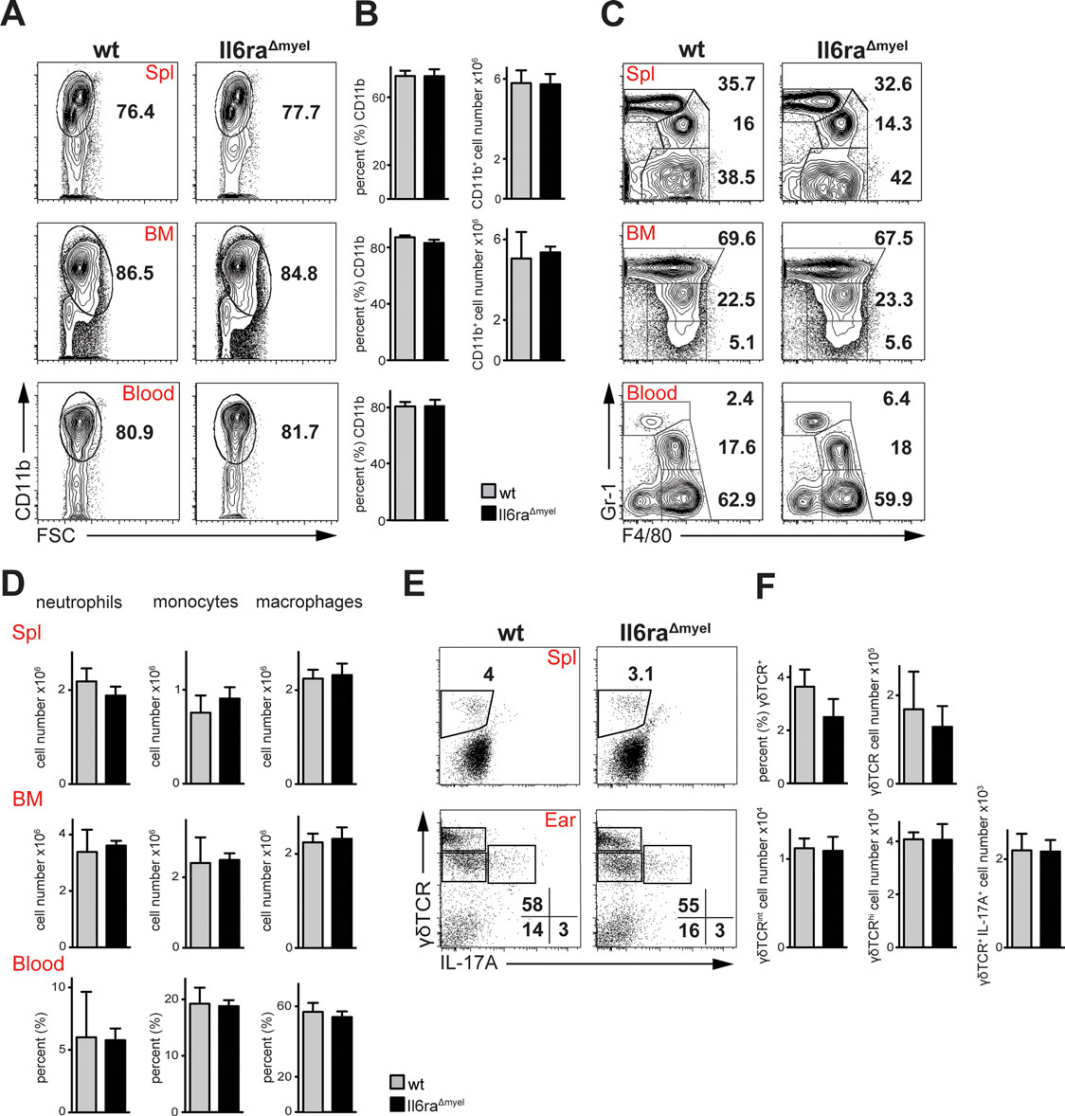
*Il6ra*^*Δmyel*^ mice show no differences in myelomonocytic cell compartments and T cells compared to *wt*. (A) Flow cytometric analysis of CD11b^+^ cells of the indicated organs. Cells are pre-gated on living CD90.2^-^/CD19^-^ cells (n = 5). (B) Percentages and total cell numbers of CD11b^+^ cells of the indicated organs. Bar graphs are shown with mean and SEM. Significance was calculated using Mann Whitney test (n = 5). (C) Flow cytometric analysis of Gr-1^+^ and F4/80^+^ cells of the indicated organs. Cells are pre-gated on living CD90.2^-^/CD19^-^ and CD11b^+^ cells (n = 5). (D) Total cell numbers of neutrophils (Gr-1^hi^ F4/80^-^), monocytes (Gr-1^int^ F4/80^+^) and macrophages (Gr-1^-^ F4/80^+^). Data are shown as bar graphs with mean and SEM. Significance was calculated using Mann Whitney test (n = 5). (E) Flow cytometric analysis of IL-17A producing γδ T cells of indicated organs. Cells are pre-gated on living CD45.2^+^/CD3^+^ cells (n = 5). (F) Total cell numbers of γδ TCR^+^ and IL-17A^+^ γδ TCR^+^. Data are shown as bar graphs with mean and SEM. Significance was calculated using Mann Whitney test (n = 5).

We analyzed if the lack of IL-6Rα in the myelomonocytic lineage has an effect on various immune cell populations in different organs. In steady state conditions flow cytometric analysis revealed that the CD11b^+^ population was not affected in spleen, bone marrow and blood of *IL-6Rα*^*Δmyel*^ compared to *wt* mice (Fig 2A and 2B). Furthermore, there was no difference in cell numbers and ratios of distinct myelomonocytic populations between IL-6R deficient and control mice (Fig 2C and 2D). Also, flow cytometric analysis of spleen and lymph nodes showed no differences in subpopulations of B and T cells (data not shown).

**Fig 3:**
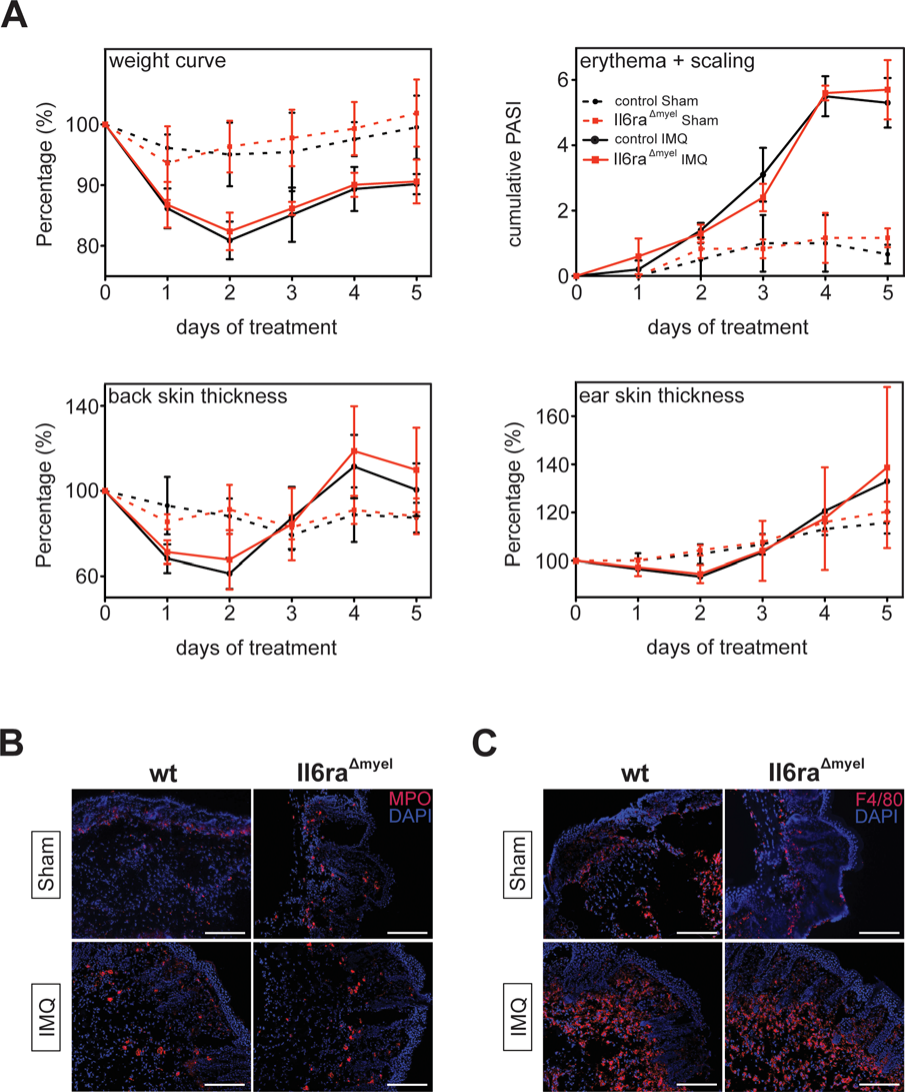
*Il6ra*^*Δmyel*^ mice show no effect in clinical scores and histology in IMQ-induced psoriasis compared to *wt* mice. (A) Weight, ear/back skin thickness and erythema/scaling was scored daily on a scale from 0 to 4 with a modified PASI from human. IMQ treated groups with n = 6, sham treated group n = 3. (B) Back skin of *Il6ra*^*Δmyel*^ and *wt* mice treated with sham (show one representative) or IMQ (n = 5) was stained by fluorescence-Immunohistochemistry for myeloperoxidase (MPO)^+^. (C) Back skin of *Il6ra*^*Δmyel*^ and *wt* mice treated with sham (show one representative) or IMQ (n = 5) was stained by fluorescence-Immunohistochemistry for F4/80^+^. (magnifications are given from representative stainings), white scale bars = 200μm.

The importance of IL-17A in IMQ-induced psoriasis-like skin disease is undisputed and the main producers of IL-17A in this model are γδ T cells [8,10]. In the skin, two distinct populations of γδ T cells can be observed, dermal (γδ TCR^lo^) and epidermal γδ T cells (γδ TCR^hi^), whereas the dermal γδ T cells have been described to be the main producers of IL-17A [8,10]. To examine the levels of IL-17A-producing γδ TCR^+^ cells in the *IL-6Rα*^*Δmyel*^ mouse line, we performed flow cytometric analysis of spleen and ear skin (Fig 2E). We also detected two populations of γδ T cells in the skin where the dermal γδ T cells clearly producing IL-17A (Fig 3E), but no differences in the expression of γδ TCR^+^ (spleen and ear) either in number of IL-17A^+^ γδ TCR^+^ (ear) cells in comparison to control animals in untreated conditions (Fig 3F).

### A prohibited IL-6 signaling pathway in myelomonocytic cells does not attenuate the development of IMQ-induced psoriasis-like skin disease

As IL-6 has a central role in the development of psoriasis, this cytokine could serve as a potential target in the treatment of this disease [15]. We therefore analyzed the IL-6-mediated signaling in myelomonocytic cells in psoriasis and treated back and ear skin of 7–8 weeks old *IL-6Rα*^*Δmyel*^ mice and the respective control groups with IMQ or sham cream for 5 consecutive days [6]. The severity of disease was documented with the Psoriasis Area and Severity Index (PASI) system, which is adapted from the severity index used for psoriasis patients [9]. Following the treatment with IMQ, back skin thickness and the cumulative PASI of erythema and scaling increased with days of treatment in comparison to sham-treated mice. However, measured parameters between *IL-6Rα*^*Δmyel*^ and *wt* mice were the same (Fig 3A). Likewise, we could not detect any difference in the ear skin thickness among IMQ treated groups showing no clinical effect of the IL-6Rα deletion on disease progression (Fig 3A).

We examined the back skin of the four different groups for infiltrating cells of the myelomonocytic compartment via fluorescent immunohistochemistry after 5 days of treatment. Skin thickness of IMQ treated mice seemed to be increased compared to sham treated groups (Fig 3B and 3C). IMQ-treatment resulted in more neutrophils (MPO^+^ cells) (Fig 3B) and more macrophages (F4/80^+^ cells) infiltrating the skin (Fig 3C) in both IMQ treated groups compared to sham treated mice, although there were no differences in infiltrating cells within the IMQ-treated groups visible.

### Myelomonocytic-specific IL-6 signaling does not affect immune cell infiltration in IMQ-induced psoriasis

**Fig 4:**
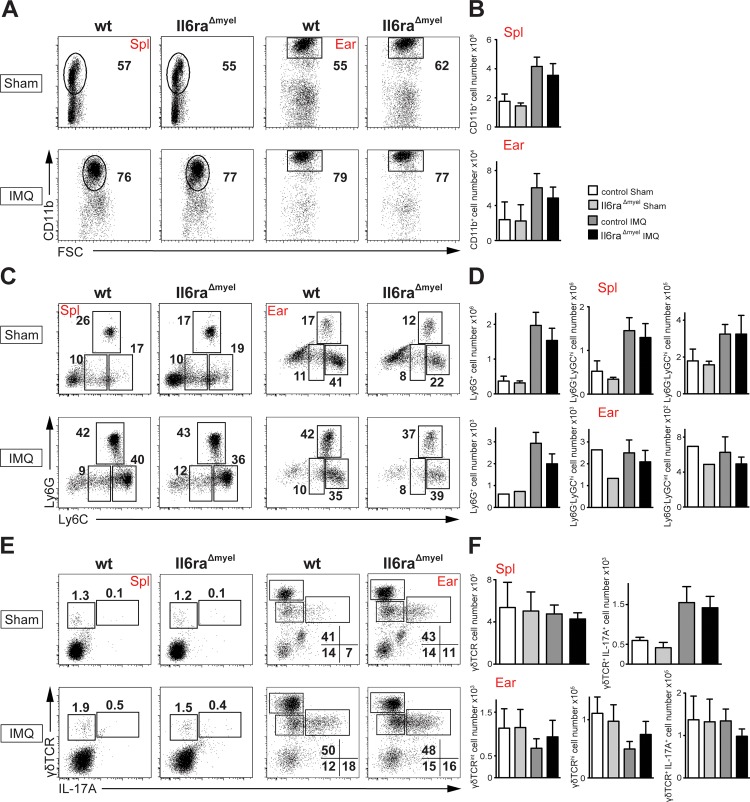
No differences of *Il6ra^Δmyel^* mice in myelomonocytic cells and T cells compared to *wt* in IMQ-induced psoriasis. (A) Flow cytometric analysis of CD11b^+^ cells of the indicated organs. Cells are pre-gated on living CD90.2^-^/B220^-^ cells. (B) Total cell numbers of CD11b^+^ cells of the indicated organs. Bar graphs are shown with mean and SEM. Significance was calculated using Kruskal-Wallis test. (C) Flow cytometric analysis of Ly6C^+^ and Ly6G^+^ cells of the indicated organs. Cells are pre-gated on living CD90.2^-^/B220^-^ and CD11b^+^ cells. (D) Total cell numbers of Ly6G^+^ cells, pro-inflammatory monocytes (Ly6G^-^Ly6C^hi^) and resident monocytes (Ly6G^-^Ly6C^int^). Bar graphs of the indicated organs are shown with mean and SEM. Significances of the spleen were calculated using Kruskal-Wallis test (IMQ groups n = 6, sham groups n = 3). Significances of the ears were calculated with Student’s t-Test (IMQ groups n = 6, sham groups n = 1). (E) Flow cytometric analysis of IL-17A producing γδ T cells of the indicated organs. Cells are pre-gated on living CD45.2^+^/CD3^+^ cells. (F) Total cell numbers of γδ TCR^+^ and IL-17A^+^ γδ TCR^+^. Data are shown as bar graphs with mean and SEM. Significance was calculated using Kruskal-Wallis test.

Treatment of murine skin with IMQ results in a different profile and increased numbers of infiltrating cells in the skin and secondary lymphoid organs [6,9,31]. Flow cytometric analysis of spleen and ears showed more CD11b^+^ cells after IMQ treatment compared to sham-treated mice (Fig 4A and 4B). Analysis of the lymph nodes gave the same result (data not shown).

Analyzing the sub-populations of CD11b^+^ cells in spleen and ears using Ly6G and Ly6C markers for differentiation of neutrophils (Ly6G^+^), pro-inflammatory monocytes (Ly6G^-^Ly6C^hi^) and resident monocytes (Ly6G^-^Ly6C^int^) [33], we found all sub-populations increased under IMQ treatment compared to sham treated groups in the spleen (Fig 4C and 4D). The ears showed a different picture, as the sham treated controls seem to have the same amount or monocytes as the IMQ treated groups. It was already shown that steric acids as a component of the sham cream can already lead to infiltration of cells [34]. There were no differences in invading Ly6G^+^ / Ly6C^+^ cells under IMQ-treatment in *IL-6Rα*^*Δmyel*^ mice compared to *wt* mice in spleen and ears (Fig 4C and 4D) [34].

In total, this was in line with the fact that the clinical appearance was not significantly altered in mice providing IL-6Rαdeletion in myelomonocytic cells–with and without IMQ-treatment.

Pantelyushin et al. had shown an increase in numbers of IL-17A producing γδ T cells under IMQ treatment [10]. FACS plots and total cell numbers of the spleen show comparable γδ TCR^+^ cells in all four groups (Fig 4E and 4F). However, total IL-17A - producing γδ T cells in IMQ-treated were increased (Fig 4F), against the pictures the FACS plots showed. However, within IMQ treated groups of the spleen no differences were detectable.

In the ears, percentages of the two γδ TCR^+^ populations were comparable in all groups (Fig 4E). However, in total cell numbers dermal and epidermal γδ T cells were elevated in sham treated groups. Probably due to more dermal γδ T cells in sham-treated groups, IL-17A-producing γδ T cells were comparable in the ears of IMQ-treated mice and sham-treated groups (Fig 4E and 4F). Altogether, in the ears the effect was not so intense as under IMQ treatment γδ T cells in the skin peak after 7 days of treatment [31,35].

Our study finally demonstrates that the myelomonocytic-specific deletion of IL-6 signaling has no effect on the distribution of immune cell populations, including B and T cells as well as myelomonocytic cells. In the model of IMQ-induced psoriasis-like skin disease, the prohibited IL-6 signaling pathway in myelomonocytic cells has no influence on the development and the severity of the disease, according to the clinical appearance and the number of infiltrating cells in the skin and secondary lymphoid organs.

## Discussion

The application of IMQ leads to a strong recruitment of T cells and myelomonocytic cells, like neutrophils, monocytes and macrophages to the site of inflammation [31]. IL-6 is of great importance for the recruitment of neutrophils in general [24] and it is associated with psoriatic skin lesions [26]. Previous studies suggested that IL-6 signaling plays an important role in the development of IMQ-induced psoriasis-like skin disease [6,9] and in spontaneous psoriasis [27,36]. In this study, we deleted the IL-6Rα specifically in the myelomonocytic cells, expecting these mice to develop a milder form of disease–in order to further investigate the role of IL-6 in the context of IMQ-induced psoriasis-like skin disease. For deletion of the IL-6Rα in myelomonocytic cells, we used the LysM-Cre, which is a Cre-recombinase of high efficiency for neutrophil granulocytes (nearly 100%) and somewhat lower efficiency in macrophages (83%-98%) [30]. Other groups already demonstrated a good depletion capacity of this Cre line in macrophages, as shown in the LysM-Cre/iDTR model, where 95% oft the macrophages could be depleted upon diphtheria toxin injection [28]. Furthermore, a recent paper that focused on the *IL-6Rα*^*Δmyel*^ mice in the context of obesity showed that in bone marrow derived macrophages of these mice no pSTAT3 could be detected by Western blot, underlying a high recombination potential of the LysM-Cre also in macrophages [37]. In contrast to our initial expectations, the removal of IL-6Rα in myelomonocytic cells [17] had no effect on the severity of IMQ-induced psoriasis-like skin disease: The *IL-6Rα*^*Δmyel*^ mice presented the same PASI scores for skin thickness, erythema and scaling as the control animals under IMQ-treatment. In line with that, the numbers of neutrophils, monocytes, macrophages and IL-17A producing γδ T cells in the skin as well as in secondary lymphoid organs in IMQ treated *IL-6Rα*^*Δmyel*^ were comparable with those in IMQ treated *wt* mice. So, the inflammatory cell invasion to the skin accompanied by the clinical signs of psoriasis was not at all reduced when the IL-6Rα in myelomonocytic cells was deleted. It is plausible that this could be at least partially due to the alternative pathway in IL-6 signaling which is one of the 2 forms of IL-6 signaling. One is the signaling of IL-6 via the mIL-6Rα and gp130. Besides this, there is the so-called IL-6 trans-signaling conveyed through the soluble form of IL-6Rα [22]. It was previously shown that the mIL-6Rα is only expressed by hepatocytes, neutrophils, monocytes/macrophages and some leukocytes [38]. Under deletion of mIL-6Rα in monocytes/macrophages and granulocytes in the *IL-6Rα*^*Δmyel*^ mice, the hepatocytes and some leukocytes could still be the source of sIL-6Rα for trans-signaling via the proteolysis of ADAM 10 or ADAM 17. There was indeed significant reduction of the sIL-6Rα in *IL-6Rα*^*Δmyel*^ mice compared to *wt* mice, but only of 25% leaving potentially enough sIL-6Rα for triggering the inflammatory cascade by initiating trans-signaling.

We noted a significant reduction of the IL-6Rα in the *IL-6Rα*^*Δmyel*^ mice (shown by RT-PCR of CD11b^+^ purified bone marrow cells and by FACS analysis of the different myelomonocytic populations. This confirms the above-mentioned efficient recombination of the LysM-Cre. Further, the analysis of CD11b^+^ stimulated splenocytes revealed a strong reduction of STAT3 in *IL-6Rα*^*Δmyel*^ mice (data not shown). Probably this result can be explained by a feedback loop induced by SOCS proteins [39]. The signal through the mIL-6Rα activates the JAK/STAT3 pathway [40] [41]. Interestingly, mice with constitutively active STAT3 in keratinocytes, develop a psoriasis like phenotype [32].

The involvement of myelomonocytic cells in IMQ-induced psoriasis-like skin disease is undisputed [6] and IL-6 as neutrophil-recruiting cytokine is known to be strongly associated with psoriasis [3]. Recently, we demonstrated that IL-6 is upregulated in IMQ-induced psoriasis-like skin disease [9]. Mice, that spontaneously develop psoriasis-like disease based on IL-17A overexpression in the skin (*K14-IL-17A*^*ind/+*^), show at least a partial remission of disease after anti-IL-6 treatment [27]. Furthermore, Il-6 signaling on keratinocytes seems to be important in the context of psoriasis. Also non-myelomonocytic cells, like the keratinocytes express a functional IL-6Rα [42] and are involved in the immune response. Their release of chemokines can also contribute to the recruitment of myelomonocytic cells to the skin dependent on JunB/AP-1 [43]. Also, a functional IL-6Rα can be shedded from these cells and therefore induce trans-signaling on myelomonocytic cells in the skin.

IL-6 deficient mice show reduced skin inflammation after intradermal injection of IL-23 [14], which is a strong trigger of erythema and inflammatory infiltrates of the skin [12,13]. As we did not see a difference in the distribution of myelomonocytic cell populations within the IMQ treated mice groups, although the mIL-6Rα is efficiently depleted in granulocytes and monocytes/macrophages, we assume that deletion of mIL-6Rα is compensated by signaling mediated via sIL-6Rα [22]. General IL-6 blockade might lead to a clinical improving effect–but the deletion of the IL-6Rα on the myelomonocytic cells does not.

In the recent years, it was discovered that, besides the myelomonocytic compartment, the IL-17/IL-23/IL-22 axis is also relevant in the IMQ-induced psoriasis-like skin disease [6,8,10,44–46]. In addition to myelomonocytic cells, γδ T cells also invade to the skin. Dermal γδ T cells are the main producers of IL-17A [8,10] and IL-22 [47] in IMQ-induced psoriasis-like skin disease. We could also show that dermal γδ T cells represent a dominant IL-17A-producing population in IMQ-treated skin. There was a prominent decrease in the number of γδ T cells in the ear skin of IMQ—treated group in comparison to sham control. This can be explained by the fact that the accumulation of γδ T cells in the skin peaks after 7 days of treatment with IMQ [31,35] and not after 5 days when we analyzed the γδ T cells in our mice. It is known that γδ T cells express the chemokine receptor CCR6 and through its expression they can be recruited to the skin by keratinocytes and dendritic cells [48]. Besides, it was reported that IL-23 induces CCR6^+^ γδ T cells [46] through the administration of IL-23 to the mouse skin [49]. As IL-6^-/-^ mice have been described to develop reduced skin disease after administration of IL-23 [14], IL-23 seems to be the connection between IL-6 and the γδ T cells in psoriasis. Additionally, it was already pointed out that IL-6 is essential for myelomonocytic cells in acute inflammation [50]. As we also did not see a difference in the expression of γδ T cells within the IMQ treated wt mice and the IMQ-treated mice lacking the IL-6 in the myelomonocytic cells, it is again likely that the trans-signaling of IL-6 compensates the missing mIL-6Rα in myelomonocytic cells. So, focusing exclusively on the classical IL-6 signaling might not be an ideal treatment option in psoriasis.

Taken together, the deletion of IL-6Rα in the myelomonocytic compartment did not affect the development of IMQ-induced psoriasis-like skin disease. Thus, IL-6 signaling must be compensated by other signaling cascades in IMQ-induced psoriasis—as the trans-signaling of IL-6. This should be kept in mind in the development of new treatment options in human psoriasis.
